# New Evidence Should Inform WHO Guidelines on Multiple Micronutrient Supplementation in Pregnancy

**DOI:** 10.1093/jn/nxy279

**Published:** 2019-02-15

**Authors:** Christopher R Sudfeld, Emily R Smith

**Affiliations:** 1 Department of Global Health and Population, Harvard TH Chan School of Public Health, Boston, MA; 2 Bill & Melinda Gates Foundation, Seattle, WA

**Keywords:** micronutrient supplementation, pregnancy, low birth weight, infant mortality, World Health Organization

## Abstract

Recent data from an individual patient data (IPD) meta-analysis of 17 randomized control trials including >100,000 women living in low- and middle-income countries found that multiple micronutrient supplementation (MMS) in pregnancy reduced the risk of low birth weight, preterm birth, and being born small for gestational age. Further, MMS reduced the risk of neonatal and infant mortality for females, and there was no evidence of increased risk among the 26 subgroups examined. The 2016 WHO antenatal care guidelines, which were released before the IPD meta-analysis, did not universally recommend MMS, noting: “There is some evidence of additional benefit … but there is also some evidence of risk.” The guidelines suggest that MMS may increase the risk of neonatal mortality based on an exploratory subgroup analysis of 6 randomized trials. However, we identified several issues with this subgroup analysis. In this report we correct and update the subgroup analysis and show that there is no evidence that MMS increases the risk of neonatal mortality. There is growing scientific consensus that MMS containing iron and folic acid (IFA) is superior to IFA alone. The WHO guidelines currently state that “policy-makers in populations with a high prevalence of nutritional deficiencies might consider the benefits of MMN [multiple micronutrient] supplements on maternal health to outweigh the disadvantages, and may choose to give MMN supplements that include iron and folic acid.” This equivocal guidance has created confusion about the best course of action for public health programs in low- and middle-income countries. Given the new evidence, WHO should review their statements regarding the potential neonatal mortality risks and re-evaluate the overall potential benefits of implementing MMS as a public health program.

Micronutrient deficiencies in pregnancy are common and are associated with adverse birth outcomes (1). Prenatal multiple micronutrient supplementation (MMS) can improve outcomes, and our recently published individual patient data (IPD) meta-analysis showed that MMS decreased mortality for female neonates and provided greater reductions in the risk of low birth weight and preterm birth for infants born to undernourished and anemic women (2).

The 2016 WHO antenatal care guidelines do not universally recommend MMS, noting: “There is some evidence of additional benefit of MMN [multiple micronutrient] supplements containing 13–15 different micronutrients (including iron and folic acid) over iron and folic acid supplements alone, but there is also some evidence of risk” (2, 3). The guidelines suggest that MMS may increase the risk of neonatal mortality based on an exploratory subgroup analysis of 6 randomized trials that used an iron-folic acid (IFA) control consisting of 60 mg Fe/d and 400 µg folic acid/d. There was a nonsignificant elevated risk of neonatal mortality in this subgroup (RR: 1.22; 95% CI: 0.95, 1.57) (3). The rationale for this exploratory analysis, and the reason for excluding trials based on the folic acid dose, were not stated.

Given the seemingly disparate findings between the recent IPD meta-analysis and the WHO subgroup analysis, we compared the methods and estimates in the published meta-analyses. We identified 4 issues in the neonatal mortality subgroup meta-analysis in the WHO guidelines, and we made the following changes: *1*) corrected the Bhutta et al. estimate, which is consistent with the updated 2017 Cochrane Review (4); *2*) included the omitted MINIMat study (5); *3*) added 2 recently published trials (lipid-based supplements arms excluded) (6, 7); and *4*) included 2 trials that used a 60 mg Fe/d control but were excluded due to using a 250 µg/d folic acid dose (2, 8, 9). The Bhutta et al. trial estimate for neonatal mortality was incorrectly presented in the 2015 Cochrane Review (RR: 0.97; 95% CI: 0.66, 1.45) (10) and was corrected in the 2017 Cochrane Review (RR: 1.44; 95% CI: 0.95, 2.18) (4). The 2017 Cochrane Review estimate is consistent with the data presented in the main trial report which includes singleton and multiple births; we used this estimate (4, 11). The Fawzi et al. (8) trial was not included in the 2015 Cochrane Review or WHO subgroup analysis of neonatal mortality because the main trial report only presented the effect of MMS on 6-wk infant mortality (4). Here we include the Fawzi et al. (8) neonatal mortality estimate as published in the IPD meta-analysis (2). The MINIMat estimate was calculated from the main trial report (see **Figure 1** footnote for calculation details) (5). After making these changes and updates, the pooled RR of neonatal mortality was 1.05 (95% CI: 0.85, 1.30) based on data from 11 trials (5–9, 11–16), and we conclude there is no indication that MMS increases the risk of neonatal mortality in the subgroup of trials that used a 60-mg Fe control group (Figure 1).

**FIGURE 1:**
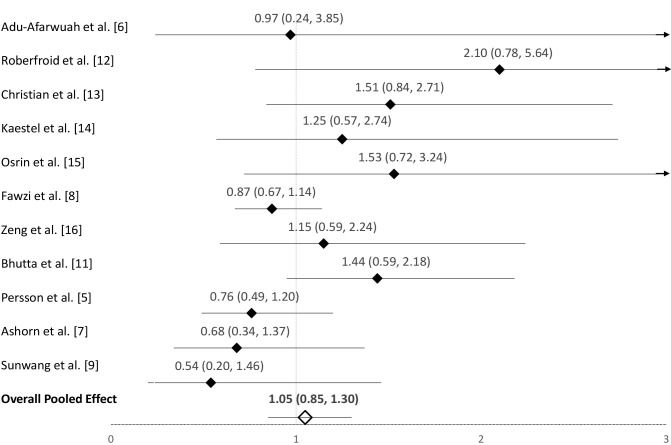
Forest plot for the effect of MMS on neonatal mortality among trials including a 60-mg Fe control group. Data are presented as RRs (95% CIs). ^1^MINIMat RR calculation: MMS group = 32 neonatal deaths out of 1190 live births including twins. Control group (60 mg Fe/d and folic acid) = 43 neonatal deaths out of 1222 live births including twins. RR of neonatal mortality: 0.76 (95% CI: 0.49, 1.20). MMS, multiple micronutrient supplementation.

We hypothesize that the nonsignificant elevated risk of mortality found in the WHO guideline subgroup analysis may be related to differences in the iron dose in each trial arm. Five out of the 6 trials included in the WHO subgroup analysis compared MMS containing low-dose iron (30 mg/d) with an IFA control arm containing high-dose iron (60 mg/d). These trial designs simultaneously address 2 questions about the effect of a lower dose of iron *and* the effect of additional micronutrients. The lower dose of iron in the MMS (30 mg/d) as compared with the control arm (60 mg/d) may explain negative effect estimates seen in some trials. The 2017 Cochrane Review examined the effect of MMS on perinatal mortality stratified by iron dose in the MMS and control arms (4). Among trials that compared MMS containing 30 mg Fe with a 60-mg Fe control group, the RR of perinatal mortality was nonsignificantly elevated at 1.19 (95% CI: 0.95, 1.48), which is similar to the WHO antenatal care guidelines subgroup analysis. Whereas, the Cochrane estimate for perinatal mortality RR among trials using MMS containing 60 mg Fe compared with a 60-mg control was 1.08 (95% CI: 0.71, 1.63). Similarly, in the IPD meta-analysis there was no indication of increased risk of neonatal mortality among trials that used the same dose of iron in the MMS and IFA control arm (either 30 or 60 mg/d) with a RR of 0.96 (95% CI: 0.88, 1.04), whereas the RR of neonatal mortality for trials using 30 mg Fe in the MMS compared with 60 mg Fe in the control was 1.16 (95% CI: 0.92, 1.45) (2). Lending further support to this hypothesis, a meta-analysis of iron supplementation trials found that every 10-mg increase in iron dose linearly decreased the risk of low birth weight by 3% (95% CI: 2%, 5%) up to 66 mg (17). Although this iron meta-analysis was not completed for neonatal survival, low birth weight is an important risk factor for neonatal mortality (18). We argue that programs considering implementation of MMS in pregnancy should consider using a formulation with an iron dose similar to their current iron supplementation recommendations (i.e., MMS that contains 60 mg Fe in settings where 60 mg Fe is currently used).

The concern and related evidence that MMS may potentially increase the risk of neonatal mortality have evolved over time. Initially, reports from a trial conducted in Nepal raised concern that increased birth size due to MMS may increase the risk of cephalopelvic disproportion and neonatal asphyxia, particularly among women of short stature (13, 19). This was followed by a 2011 review by Haider et al. (20) that noted increased risk of neonatal mortality among the subgroup of trials where <60% of women delivered in a health facility (RR: 1.47; 95% CI: 1.13, 1.92). However, the recent IPD meta-analysis, which used the gold standard, individual-level, analytic approach, revealed no indication that MMS increased the risk of stillbirth or neonatal mortality among women with short statures (height <150 cm) or among women delivering without a skilled birth attendant (2). In fact, after pooling all available data, MMS was found to significantly *decrease* the risk of infant mortality for infants born to women without access to a skilled birth attendant (∼18% risk reduction) (2). The IPD analysis provides stronger causal evidence at individual level, therefore we conclude there is no evidence of harm associated with MMS and clear evidence of benefit.

Our update of the WHO subgroup analysis showed no increased risk of neonatal mortality and the results are consistent with the findings of the updated 2017 Cochrane Review and our recent IPD meta-analysis which found that MMS did not increase the risk of neonatal mortality overall or in any of 26 subgroups of pregnant women and newborns (2, 4). In fact, we found previously that MMS clearly reduces the risk of neonatal and infant mortality for female infants (2). Given the new and consistent evidence, WHO should promptly review their statements regarding the potential neonatal mortality risks associated with MMS and re-evaluate the overall benefits of implementing MMS as a public health program.

## Notes

The authors reported no funding received for this study.

Author disclosures: CRS and ERS, no conflicts of interest.

Abbreviations used: IFA, iron and folic acid; IPD, individual patient data; MMS, multiple micronutrient supplementation.
